# Prognostic Value of KRAS Mutations in Relation to PDL1 Expression and Immunotherapy Treatment in Adenocarcinoma and Squamous Cell Carcinoma Patients: A Greek Cohort Study

**DOI:** 10.3390/jpm14050457

**Published:** 2024-04-25

**Authors:** Theodora Tsiouda, Kalliopi Domvri, Efimia Boutsikou, Vasileios Bikos, Krystallia Kyrka, Konstantina Papadaki, Persefoni Pezirkianidou, Konstantinos Porpodis, Angeliki Cheva

**Affiliations:** 1 Pulmonary-Oncology Department, ‘Theageneio’ Cancer Hospital, 540 07 Thessaloniki, Greece; ttsiouda@auth.gr (T.T.); efimi_b@yahoo.gr (E.B.); vasimpik@auth.gr (V.B.); krystalk@auth.gr (K.K.); kopapadaki@uth.gr (K.P.); ppezirkianidou@uth.gr (P.P.); 2 Laboratory of Histology-Embryology, Medical School, Aristotle University, 541 24 Thessaloniki, Greece; 3 Laboratory of Pathology, “G. Papanikolaou” General Hospital, Exohi, 570 10 Thessaloniki, Greece; 4 Pulmonary Department, Medical School, Aristotle University of Thessaloniki, “G. Papanikolaou” General Hospital, Exohi, 570 10 Thessaloniki, Greece; kporpodis@auth.gr; 5 Department of Pathology, AHEPA University Hospital of Thessaloniki, Aristotle University, 541 24 Thessaloniki, Greece; antacheva@auth.gr

**Keywords:** lung cancer, KRAS, PDL1, immunotherapy, survival

## Abstract

Background: Factors that could predict which patients will benefit from Immune Checkpoint Inhibitors (ICIs) are not fully understood. This study aimed to investigate the prognostic value of KRAS biomarker in patients with advanced non-small cell lung cancer (NSCLC) in relation to clinical characteristics, treatment response and PDL1 expression. Patients and methods: The study included 100 patients with NSCLC who received immunotherapy with or without chemotherapy as 1st line treatment. In biopsy samples, the PDL1 biomarker expression rate and somatic mutations of KRAS gene were determined. Results: The mean age of the patients was 67 ± 8 years. Patients were all male and 66% were found with adenocarcinoma whereas 34% with squamous cell carcinoma. The KRAS G12C mutation was found with the highest percentage (73%). In the Kaplan-Meier survival analysis, patients with PDL1 > 49% in combination with a negative KRAS result had a median overall survival of 40 months compared to patients with a positive KRAS result (9 months, *p* < 0.05). In addition, patients diagnosed with adenocarcinoma, PDL1 < 49% and negative KRAS result had a median overall survival of 39 months compared to patients with a positive result (28 months, *p* < 0.05). Conclusions: Our study suggests that the presence of KRAS mutations in advanced NSCLC patients has a poor prognostic value, regardless of their PDL1 expression values, after receiving immunotherapy as first-line treatment.

## 1. Introduction

According to global cancer burden using the GLOBOCAN 2020, estimating cancer incidence and mortality produced by the International Agency for Research on Cancer, lung cancer is still the second most common cancer, after breast cancer [1]. Recently, the development of immunotherapy based on the theory that the immune system plays a protective role by recognizing cancer cells has led to considerable progress in the treatment of non–small-cell lung cancer (NSCLC) with a substantial reduction in mortality [2]. Despite the benefits that have been established with immunotherapy in the metastatic setting, there are still many patients that do not benefit from this treatment regardless the PDL1 expression status, indicating the need to identify novel predictive factors [3,4]. In addition, spatial and temporal heterogeneity of the solid tumor is the main obstacle for consistency in PDL1 testing [5]. Currently, PDL1 expression remains a predictive biomarker for response to single-agent immunotherapy, to median progression free survival, to median overall survival and clinical outcome. Besides, the efforts to better identify cancer patients have led to the approvals of targeted therapies for patients with specific oncogenic driver mutations. Checkpoint inhibitors have also been improved for patients without an actionable driver mutation given either as monotherapy or in combination with chemotherapy. However, the prognosis for advanced NSCLC patients remains unsatisfactory, with a median progression-free survival of only 2 to 4 months associated with chemotherapy agents or checkpoint inhibitors [6]. Predictive and prognostic biomarkers are at need to help physicians determine subgroup of patients who may benefit from specific treatments.

Concerning the targeted therapies, the most prevalent genomic driver event in NSCLC is activating mutations in Kirsten rat sarcoma viral oncogene homologue (KRAS) in about 25 to 30% of non–squamous-cell NSCLCs in Western countries [7]. The most common KRAS mutation subtype is the G12C mutation, which accounts for approximately 13% of all KRAS mutations in NSCLC [8]. Other less common KRAS mutation subtypes include G12D, G12V, G13C, and Q61H/L which have been implicated with differences in the prognosis and response to treatment in KRAS positive patients [9,10]. KRAS mutations are found with different frequencies in males than in females and vary also according to patient ethnicity [11] and in specific they are more common in Western versus Asian patients (26% versus 11%) [12]. In addition, they are more commonly found in patients with a history of smoking (30% versus 11%) [13]. Furthermore, it has been reported that KRAS mutations are more common in adenocarcinoma (20–40%) and less common in squamous cell carcinoma (<5%) [14].

Overall, KRAS mutations constitute a distinct NSCLC subset of patients as KRAS mutations where standard of care provides modest clinical benefit. It has also been demonstrated that KRAS mutations are mutually exclusive with other oncogene mutations such as epidermal growth factor receptor (EGFR) mutations [15]. Several meta-analyses have tried to elucidate the prognostic value of KRAS mutation in patients with advanced NSCLCs, however with inconsistent results [15,16,17]. Thus, there is a need to further clarify the role of KRAS mutations in overall survival of NSCLC patients.

The aim of the current retrospective cohort study was the investigation of the role of KRAS mutational status as predictive biomarker on overall survival (OS) and its relation to PDL1 expression and other clinical characteristics of patients diagnosed with advanced NSCLC after receiving immunotherapy with or without platinum-based chemotherapy.

## 2. Patients and Methods

### 2.1. Study Cohort

This is a monocenter retrospective study of patients followed up in Pulmonary-Oncology Unit of ‘Theageneio’ Cancer Hospital in Thessaloniki, approved by the Local Ethics Committee of the same Hospital. We included 100 patients with pathologically documented with locally advanced or metastatic NSCLC according to World Health Organization classification and guidelines [18], who had disease progression after the receipt of anti–programmed death 1 (PD-1) immunotherapy (pembrolizumab, nivolumab, ipilimumab and atezolizumab) or after the receipt of both immunotherapy and platinum-based chemotherapy according to ASCO guidelines [19,20]. Response assessment was performed after 2 cycles, then every 2–4 cycles with CT of known or high-risk sites of disease with or without contrast. Patients enrolled in the analysis had either no adverse events due to immunotherapy or adverse events grade 1 according to the Common Terminology Criteria for Adverse Events (CTCAE) v5.0. and their performance status was between 0–2.

### 2.2. Genetic Analysis and Immunohistochemistry Analysis

Molecular testing of somatic mutations of the KRAS gene was retrospectively performed with Real-Time PCR technology for each NSCLC patient. KRAS Mutation Test (KRAS-RT50 CE-IVD kit) by EntroGen, Incorporation in the system of Applied Biosystems ABI 7500 was used, able to investigate 16 mutations in exons 2, 3, 4 of KRAS gene [21]. PDL1 expression was assessed in 5-μm sections cut from formalin-fixed and paraffin-embedded NSCLC tissue blocks using an anti-PDL1 antibody (clone IHC411, GenomeMe, Richmond, Canada) on the LEICA BOND-III platform. Stained slides were then observed under a light microscope to assess positivity. Two pathologists blindly calculated both biomarkers for interobserver and intraobserver variability. The PDL1 tumor proportion score (TPS) was calculated as the percentage of at least 100 viable cancer cells with complete or partial membrane staining. Necrotic areas were excluded from scoring. PDL1 positivity and PDL1 high expression, was defined as staining in ≥1% and ≥49% of all tumor cells, as used in other clinical trials [22]. No differences were reported among calculations of the two pathologists.

### 2.3. Statistical Analysis

Sample size was calculated using the G*Power software v 3.1.9.4 (Die Heinrich-Heine-Universität Düsseldorf, Düsseldorf, Germany). A two-sided test, with a power at 0.986 and an effect size of 0.5 indicated a total sample size of 100 participants. Statistical analysis was performed using the SPSS (version-21.0 IBM-SPSS-statistical-software, Armonk, NY, USA). Descriptive statistics were performed, and quantitative data were summarized as means with standard deviation (SD). Qualitative variables were summarized as frequencies in the entire population and percentages. Shapiro–Wilk test was performed for normality test. The differences between groups regarding KRAS mutational status, NSCLC histology or PDL1 expression cut-off points were determined with a two-tailed Student’s *t*-test and Mann–Whitney U test for non-parametric variables. Kaplan-Meier curve with log-rank test was used for survival analyses regarding the comparison of the previous groups. Chi-square was used to compare groups of categorical variables regarding KRAS mutational status and PDL1 expression. Differences were considered statistically significant at *p* < 0.05.

## 3. Results

### 3.1. Clinical Characteristics

**Figure 1:**
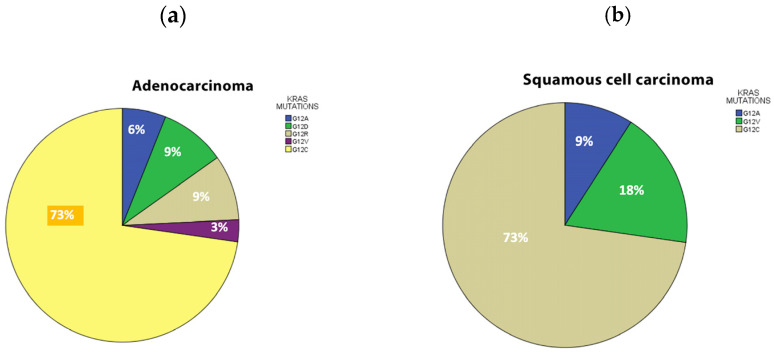
(**a**) KRAS mutational distribution in αdenοcarcinoma samples, (**b**) KRAS mutational distribution in Squamous cell carcinoma samples.

The demographics and clinical characteristics are shown in Table 1. Median overall survival was 15 months. Of the 100 NSCLC samples analyzed, 43% (*n* = 43) harbored a KRAS mutation (Table 2). The prevalence of KRAS mutations was 43% (*n =* 33) among adenocarcinoma (Table 2) and 29% (*n =* 10) among squamous cell carcinoma samples. The smoking status was categorized as never smokers (4%), light smokers (3%) (designated as less than 15 packs per year) and current smokers (93%). Among KRAS mutation status, 46% of current smokers were found positive (Table 2). No statistically significant differences were found in patients with positive KRAS results, among the histology subtypes in relation to clinical characteristics (Table 3). The most common was G12C (72%) followed by the others with of 7% each (G12A, G12D, G12R, G12V) with no statistically significant differences in relation to age and OS (Table 4). KRAS mutational distribution in all NSCLC histology samples appear in Figure 1. No statistically significant difference was found in age between KRAS subtypes.

### 3.2. Patients’ Survival Regarding KRAS Mutational Status in Relation to NSCLC Histology

**Figure 2:**
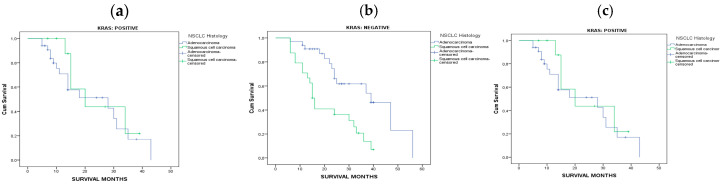
(**a**) Kaplan-Meier survival curves for patients diagnosed with adenocarcinoma in relation to KRAS mutational status, (log-rank *p* value: 0.005) (**b**) Kaplan-Meier survival curves for patients with negative KRAS status, diagnosed with adenocarcinoma in relation to patients with squamous cell carcinoma, (log-rank *p* value: 0.001), (**c**) Kaplan-Meier survival curves for patients with positive KRAS status, diagnosed with adenocarcinoma in relation to patients with squamous cell carcinoma, (log-rank *p* value: 0.501).

Concerning overall survival, statistical significance was found in Kaplan-Meier survival curves for patients diagnosed with adenocarcinoma in relation to KRAS mutational status, (log-rank *p* value: 0.005) (Figure 2a). Median overall survival of patients with adenocarcinoma found positive with KRAS mutation was statistical significantly lower when compared to patients with negative KRAS mutation status (25.3 vs. 15.1, *p* < 0.001) (Table 2). In addition, statistical significance was found in Kaplan-Meier survival curves for patients with negative KRAS status, when comparing those diagnosed with adenocarcinoma in relation to patients with squamous cell carcinoma, (log-rank *p* value: 0.001) (Figure 2b), whereas no statistical significance was found in Kaplan-Meier survival curves for patients with positive KRAS status (log-rank *p* value: 0.501) (Figure 2c). Overall, patients with negative KRAS results, diagnosed with adenocarcinoma were found with better survival compared to those with squamous cell carcinoma (25.3 vs. 19.4 months) (Table 2).

### 3.3. PDL1 Expression in Relation to KRAS Mutational Status and Clinical Characteristics

**Figure 3:**
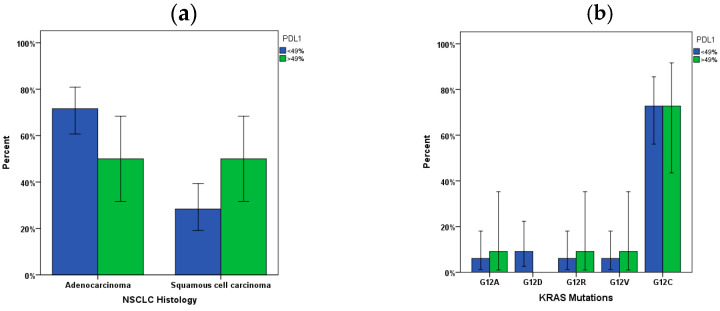
(**a**) Distribution of PDL1 expression across NSCLC histology subtypes (*p* < 0.05), (**b**) Frequency of KRAS mutation subtypes in PDL1 expression cut offs (Error bars: 95% CI).

**Figure 4:**
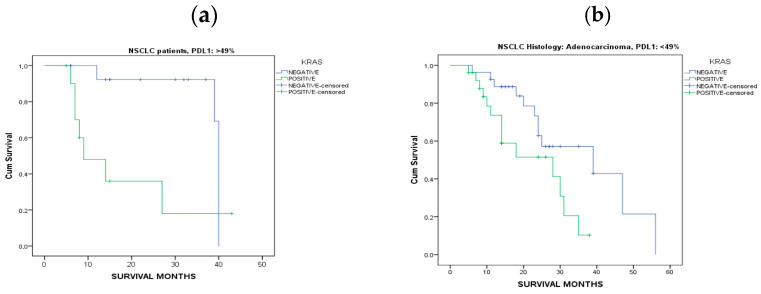
(**a**) Kaplan-Meier survival curves for NSCLC patients with PDL1 expression > 49%, (log-rank *p* value: 0.013) (**b**) Kaplan-Meier survival curves for patients diagnosed with adenocarcinoma with PDL1 expression < 49%, (log-rank *p* value: 0.023).

PDL1 expression was found >49% in 26% of NSCLC patients as appeared in Table 1. PDL1 expression values were distributed across NSCLC histology subtypes with statistical significance (*p* = 0.045). Eleven patients with positive KRAS mutations were found with high values of PDL1 expression (>49%) (Table 2). Higher percentages of patients with PDL1 expression < 49% were found in patients diagnosed with adenocarcinoma (Figure 3a). Additional analysis demonstrated that there were no significant differences between PDL1 expression and KRAS mutation subtypes (Figure 3b). Furthermore, no statistically significant difference was found among patients’ response after 6 months of immunotherapy treatment in relation to PDL1 expression and KRAS mutational status (Table 5). The higher percentage (20%) of NSCLC patients with progressive disease after 6 months of treatment was found in patients with positive KRAS mutational status with PDL1 expression < 49%. In addition, 12% of our cohort with positive KRAS result, were found with partial response, complete response, or stable disease. Besides, Kaplan-Meier survival analysis was performed and patients with PDL1 expression > 49% and negative KRAS mutation were found with median overall survival of 40 months when compared to patients with positive KRAS mutation (9 months, *p* < 0.05), (Figure 4a). Also, Kaplan-Meier survival analysis in patients with PDL1 expression < 49% diagnosed with adenocarcinoma and negative KRAS mutation were found with median overall survival of 39 months when compared to patients with positive KRAS mutation (28 months, *p* < 0.05), (Figure 4b). As the expression threshold defining positive and negative PDL1 expression is also widely debated, to perform more comparisons in different subgroups, threshold of 1% was also used. However, no significant differences were reported at threshold of 1% as a cutoff point concerning PDL1 expression.

## 4. Discussion

As the prevalence of KRAS mutations in lung cancer is high, there has been significant interest in developing targeted therapies that specifically address these mutations. Although preclinical and clinical studies have provided encouraging data, there is a notable variation in treatment outcomes, response duration, and resistance mechanisms [23]. Such variations may be influenced by specific co-mutations that are present at the time of diagnosis [24]. Due to this inconsistent response to therapy, the development of effective treatments for lung cancer patients with KRAS mutations remains a challenge. Therefore, there is a need for prognostic biomarkers that can help physicians identify a subgroup of patients who may benefit from certain treatments. To our knowledge, this is the first study conducted in a Greek population that investigated the role of the mutational heterogeneity of KRAS status and its relation to PDL1 expression, as predictive biomarkers in a treated cohort. Our study suggests that the presence of KRAS mutations in advanced NSCLC patients has a poor prognostic value, regardless of their PDL1 expression values, after receiving immunotherapy as first-line treatment.

More specifically, the most significant result in our study was the identification of subgroup of patients negative to KRAS mutations regardless of PDL1 expression values that were found with statistically higher median overall survival when compared to patients with positive KRAS mutation. Our results indicate that KRAS mutational status is a biomarker of poor prognosis. Similarly, in previous studies, KRAS mutational status has been associated with a poorer prognosis in previous studies [25,26]. Additionally, in another study, researchers suggested that KRAS mutation was an independent predictor of worse overall survival in patients with lung adenocarcinoma treated with first line pembrolizumab monotherapy [27]. Besides, in a Danish cohort study, KRAS mutation status was associated with PD-L1 expression in tumors, but not with patient survival [28]. It has also been reported that the impact of KRAS mutations on prognosis may vary depending on the histologic subtype of NSCLC, the smoking history, the stage of the cancer, and the presence of other genetic mutations [29]. In the present study, 33% of patients diagnosed with adenocarcinoma were found with positive KRAS mutations whereas 10% in patients diagnosed with squamous cell carcinoma which is in accordance with previous studies reporting that KRAS mutations are more common in adenocarcinoma and less common in squamous cell carcinoma [14]. Nevertheless, no significant differences were found in patients with positive KRAS results among these two different pathologies, regarding clinical characteristics or survival. However, statistical significance was found in Kaplan-Meier survival curves for patients with negative KRAS status, demonstrating that those diagnosed with adenocarcinoma had better survival prognosis compared to patients with squamous cell carcinoma. Previous studied have already reported that squamous cell carcinoma may be a more malignant type in comparison with adenocarcinoma [30].

Furthermore, in our study, KRAS G12C was the most frequent KRAS mutation in our cohort of patients and was also the most prevalent in current smokers. Our results are in accordance with other studies [9,10,31]. Concerning immunotherapy treatment, we found no statistically significant differences among patients’ response after 6 months in relation to PDL1 expression and KRAS mutational status. According to some studies, tumors with KRAS mutations may have higher levels of PDL1 expression, suggesting that these tumors may be more responsive to immune checkpoint inhibitors [32,33]. In our study, 11 patients (11%) with positive KRAS mutation were found with high values of PDL1 expression (>49%). However, the higher percentage (20%) of NSCLC patients with progressive disease after 6 months of immunotherapy treatment with or without chemotherapy was found in patients with positive KRAS mutational status with PDL1 expression < 49%. On the contrary, recently, in a small cohort, patients with PDL1 overexpression combined with G12C mutation treated with ICIs as first line of treatment, showed significantly longer progression-free survival [27]. In addition, 12% of our cohort with positive KRAS result and PDL1 expression < 49% were reported with partial response, complete response or stable disease. Previous studies suggest that KRAS-mutant lung cancer patients with low PDL1 expression may still benefit from immune checkpoint inhibitor therapy [34]. Moreover, the results of meta-analysis confirmed that anti-PDL1 with chemotherapy or without was better than chemotherapy alone, with greater OS benefit for the subgroup with KRAS-mutant NSCLCs than wild-type KRAS tumors [16]. They suggest that anti-PDL1 therapy could be a reference therapy for evaluating the effectiveness of targeted therapies being developed for KRAS-mutant NSCLC patients, especially the subgroup with the KRAS G12C mutation. Overall, the presence of KRAS mutations may decrease the effectiveness of immunotherapy, regardless of PDL1 expression levels. This information could be useful for clinicians when deciding on the best treatment options for NSCLC patients with KRAS mutations.

One limitation of our study is the size number, to support results for genetic analysis for a population. Another limitation is that no data were available concerning different types of co-mutations that may co-exist with KRAS mutations and may contribute to worse clinical outcomes. Studies have shown that KRAS mutations often co-occur with other genetic alterations in NSCLC, including mutations in TP53 and STK11 or CDKN2A [24,35]. As co-occurring mutations may contribute to more aggressive disease and worse outcomes, understanding their occurrence could help clinicians identify patients who require more aggressive treatment strategies. Besides, it is critical to understand the mechanisms of resistance to KRAS G12C inhibitors [33]. The identification of patients at high risk, could lead to the development of more effective and durable treatment strategies for KRAS-mutant NSCLC. We also acknowledge the lack of representation of female patients with KRAS-positive NSCLC, which could be a study limitation. It is essential to note that the prevalence of KRAS mutations can also vary depending on other factors such as smoking status, histological subtype of NSCLC, and ethnicity. Therefore, while gender may play a role in mutation prevalence, it is just one of many factors to consider when analyzing genetic alterations in NSCLC.

Recently, sotorasib, formerly AMG 510, a small molecule was approved for 3rd line of treatment in patients with KRAS G12C-mutated NSCLC after previous immunotherapy and/or chemotherapy [36]. It is important to continue conducting research to better understand the impact of KRAS mutations in different treatment contexts [33]. At this concept, a study evaluated KRAS mutations in minor clones in patients with lung adenocarcinoma treated with EGFR-TKI, concluding that KRAS mutations might hinder the effectiveness of anti-EGFR therapy [37]. Furthermore, a Greek study, recently showed that the presence of KRAS mutations resulted in a significantly worse survival among patients receiving platinum-based first line treatment [38]. Future studies could explore the impact of KRAS mutations on prognosis in NSCLC patients receiving different types of treatments, such as chemotherapy or targeted therapies.

## 5. Conclusions

Our study suggests that the presence of KRAS mutations in advanced NSCLC patients has a poor prognostic value, regardless of their PDL1 expression values, after receiving immunotherapy as first-line treatment. Thus, NSCLC patients with KRAS mutations are likely to have a worse outcome, even if they are treated with immunotherapy as their first-line therapy, regardless of whether they have high or low levels of PDL1 expression. However, the relationship between KRAS mutational status and PDL1 expression remains complex and varies depending on the specific cancer type and other factors and further research is at need.

## Figures and Tables

**Table 1:** Demographics and clinical characteristics of NSCLC patients included in the study.

Characteristics	Patients (*n =* 100)
Age, mean ± SD, years	66.61 ± 8.3
Age distribution	
30–50	1% (*n =* 1)
40–50	3% (*n =* 3)
50–60	13% (*n =* 13)
60–70	41% (*n =* 41)
70–80	38% (*n =* 38)
80–90	4% (*n =* 4)
Sex, (Male/Female)	100%/0%
BMI (Mean ± SD)	25.80 ± 4.8
Smoking Status	
Never smokers	4%
Light smokers	3%
Current smokers	93%
Pack/years	77
NSCLC Histology	
Adenocarcinoma	66%
Squamous cell carcinoma	34%
PDL1 expression	
<49%	74%
≥49%	26%
Overall survival, months, Mean ± SD (Median)	19.99 ± 11.7 (15.00)

**Table 2:** KRAS mutational status distribution across NSCLC patients’ clinical characteristics.

	Negative KRAS	Positive Kras	*p*-Value (95% CI)
Patients *n =* 100	57%	43%	-
PDL1			
<49%	74% (*n =* 42)	74% (*n =* 32)	0.934
>49%	26% (*n =* 15)	26% (*n =* 11)	(1.000–1.000)
NSCLC Histology			
Adenocarcinoma	33% (*n =* 33)	33% (*n =* 33)	0.059
Squamous Cell Carcinoma	24% (*n =* 24)	10% (*n =* 10)	(0.540–0.630)
Smoking status			
Never smokers	7% (*n =* 4)	0	0.053
Light smokers	5% (*n =* 3)	0	(0.049–0.057)
Current smokers	88% (*n =* 50)	100% (*n =* 43)	
Overall survival, months, Mean ± SD, (Median)			
Adenocarcinoma	25.36 ± 11.8 (24.0)	15.18 ± 10.5 (11.0)	<0.001 * (4.67–15.68)
Squamous Cell Carcinoma	19.45 ± 11.2 (15.0)	19.40 ± 10.6 (15.0)	0.989 (−8.41–8.53)

* with statistical significant difference *p* < 0.05.

**Table 3:** Positive KRAS status distribution across adenocarcinoma and squamous cell carcinoma characteristics.

KRAS Positive *n =* 43	Adenocarcinoma *n =* 33	Squamous Cell Carcinoma *n =* 10	*p*-Values (95% CI)
Age, mean ± SD, years	68.0 ± 5.6	63.71 ± 12.1	0.267 (−13.48–4.15)
Age distribution			
40–50	0	20% (*n =* 2)	0.085
50–60	9% (*n =* 3)	10% (*n =* 1)	(0.080–0.091)
60–70	36% (*n =* 12)	40% (*n =* 4)	
70–80	55% (*n =* 18)	9% (*n =* 3)	
PDL1			
<49%	79% (*n =* 26)	60% (*n =* 6)	0.408
>49%	21% (*n =* 7)	40% (*n =* 4)	(0.398–0.418)
Response after 6 months			
PR	18% (*n =* 6)	30% (*n =* 3)	0.776
CR	9% (*n =* 3)	10% (*n =* 1)	(0.768–0.784)
SD	12% (*n =* 4)	20% (*n =* 2)	
PD	61% (*n =* 20)	40% (*n =* 4)	
Overall survival, months, Mean ± SD, (Median)	15.18 ± 10.5 (11.0)	19.45 ± 10.6 (15.0)	0.274 (−3.46–11.90)

PR: partial response, CR: complete response, SD: stable disease, PD: progressive disease.

**Table 4:** KRAS mutation subtype distribution in age and in overall survival.

KRAS Mutations	Number of Patients *n =* 44	Age Years, Mean ± SD	Median Age	*p*-Value (95% CI)	Overall Survival Months, Mean ± SD	Median Overall Survival Months	*p*-Value (95% CI)
G12A	3 (7%)	59 ± 4.5	59	0.125	17.0 ±13.1	15.0	0.215
G12D	3 (7%)	71 ± 2.6	70	(0.119–0.132)	19.66 ± 13.4	14.0	(0.209–0.221)
G12R	3 (7%)	65 ± 9.5	60		6.66 ± 1.5	7.0	
G12V	3 (7%)	68.3 ± 6	69		11.0 ± 4.0	11.0	
G12C	32 (73%)	67.6 ± 7.9	70		17.09 ± 10.6	14.0	
Total	44	66 ± 8.9	65.5		23.01 ± 11.9	22.50	

**Table 5:** Patients’ response after 6 months of treatment in relation to KRAS mutational status and PDL1 expression.

PDL1 Expression	Response after 6 Months	KRAS Mutational Status		*p*-Values (95% CI)
		Negative	Positive	
<49%	PR	11%	6%	0.324 (0.315–0.333)
	CR	5%	2%	
	SD	9%	4%	
	PD	17%	20%	
>49%	PR	5%	3%	0.978 (1.000–1.000)
	CR	3%	2%	
	SD	2%	2%	
	PD	5%	4%	

PR: partial response, CR: complete response, SD: stable disease, PD: progressive disease.

## Data Availability

The datasets used and/or analyzed during the present study are available from the corresponding author upon reasonable request.
